# Long noncoding RNAs and microRNAs in Endometriosis

**DOI:** 10.3390/biomedicines13112777

**Published:** 2025-11-13

**Authors:** Edi Muhaxhiri, Maruša Debeljak, Katarina Trebušak Podkrajšek, Helena Ban Frangež

**Affiliations:** 1 Faculty of Medicine, University of Ljubljana, Vrazov trg 2, 1000 Ljubljana, Slovenia; marusa.debeljak@kclj.si (M.D.); katarina.trebusakpodkrajsek@mf.uni-lj.si (K.T.P.); helena.ban@kclj.si (H.B.F.); 2 Department of Obstetrics and Gynaecology, University Medical Centre Ljubljana, Šlajmerjeva Ulica 3, 1000 Ljubljana, Slovenia; 3 Clinical Institute for Special Laboratory Diagnostics, University Children’s Hospital, University Medical Centre Ljubljana, Bohoričeva 20, 1000 Ljubljana, Slovenia

**Keywords:** endometriosis, long noncoding RNAs, lncRNA, microRNA, miRNA, biomarkers

## Abstract

Endometriosis represents a prevalent gynaecological disorder, impacting around 10% of the female population and affecting as many as 50% of women who are facing challenges with infertility. The pathogenesis of the disease encompasses intricate processes such as the formation of adhesions, degradation of the extracellular matrix, angiogenesis, increased cell proliferation, impaired apoptosis, and dysregulation of the immune response. Although endometriosis is common, its precise etiology remains unidentified, despite various hypotheses being suggested. Recent findings underscore the significance of non-coding RNAs, specifically long non-coding RNAs (lncRNAs) and microRNAs (miRNAs), which have been identified as important regulators in the development of endometriosis. This literature review integrates findings from various transcriptomic and molecular studies to distinguish between lncRNAs and miRNAs that are associated with direct pathogenic roles and those that simply represent altered gene expression profiles in endometriosis. Particular long non-coding RNAs, such as H19, MALAT1, and LINC01116, are associated with chromatin remodeling, functioning as competitive endogenous RNAs, and influencing critical signaling pathways. Concurrently, specific microRNAs, including the miR-200 family, miR-145, and let-7b, seem to govern processes like epithelial-to-mesenchymal transition, angiogenesis, and cell adhesion. The findings highlight the significant potential of non-coding RNAs to serve as biomarkers for diagnostic purposes and as innovative therapeutic targets. Subsequent research endeavours ought to focus on corroborating these findings and elucidating the specific pathogenic roles of these non-coding RNAs in the context of endometriosis.

## 1. Introduction

Endometriosis is a prevalent, non-cancerous gynecological condition that affects around 10% of all women and 35–50% of women who experience difficulties in conceiving [1]. It is a condition characterized by the presence of functioning endometrial-like tissue that establishes itself in an ectopic location, typically outside the uterine cavity. These cells may be situated between the uterus and rectum, between the rectum and vagina, ovaries, fallopian tubes, within the abdominal cavity, and at various sites within the body.

The prevalent manifestations associated with endometriosis encompass severe pelvic pain, dysmenorrhea; dyspareunia; gastrointestinal disturbances such as diarrhea, cramps, constipation during menstruation, nausea, and vomiting; urinary symptoms characterized by painful urinary tract issues, urge to urinate, painful pressure on the bladder, and pain during urination; as well as additional symptoms including profound fatigue, dizziness, sciatica during menstruation, radiating pain towards the breast or shoulder, and depressive symptoms. Nevertheless, a significant proportion of symptoms associated with this disease are asymptomatic, representing 20–25% [2].

The development of endometriosis involves various complex processes such as the production of adhesions, degradation of the extracellular matrix, angiogenesis, increased cell growth, reduced cell death, impaired cell communication, a changed immunological response, an altered ability to differentiate, and other abnormal physiological processes [3]. While the cause of endometriosis is uncertain, various explanations have been proposed:

- Retrograde menstruation—supported by peritoneal lesion distribution but does not explain distant sites.
- Lymphatic or hematogenous spread—underlies extra peritoneal implants, though evidence is limited.
- Coelomic metaplasia—highlights peritoneal cell plasticity, yet rarely demonstrated experimentally.
- Stem/progenitor cell theory—multipotent bone marrow and endometrial progenitor cells may differentiate ectopically.
- Müllerian remnants—embryonic rests may persist and aberrantly differentiate.

Each mechanism likely contributes variably, underscoring a multifactorial etiology [4].

Although extensive studies have been conducted on biomarkers, there is currently no blood test that can accurately and consistently detect endometriosis. The gold standard for diagnosing endometriosis has traditionally been laparoscopic visualization of lesions, confirmed by histological testing. However, recent guidelines suggest a non-surgical (clinical) diagnosis based on symptoms, physical examination results, and imaging [5,6]. Due to the non-specific symptoms and limited treatment options, it is necessary to enhance our understanding of the molecular pathogenesis of the disease. This will enable us to identify crucial factors that can serve as biomarkers for diagnosis or as targets for new treatments.

Recently, it has been shown that non-coding RNAs have a role in the development of endometriosis [7]. Non-coding RNAs are a group of RNAs that do not encode functional proteins and were first regarded as solely regulating gene expression at the post-transcriptional level.

Accordingly, this review aims to systematically synthesize and critically assess the existing evidence regarding the role of long non-coding RNAs (lncRNAs) and microRNAs (miRNAs) in the pathogenesis of endometriosis. The review seeks to integrate molecular findings and transcriptomic data, thus underscoring their significance in pathogenesis, diagnostics, and potential therapy for endometriosis.

## 2. Noncoding RNAs: Classification, Mechanisms and Functions

Non-coding RNAs play a crucial role in regulating the expression of genes that code for proteins. By doing so, they regulate the function of several signaling pathways. They are classified into two primary categories based on their length: short-chain non-coding RNAs (including siRNAs, miRNAs, and piRNAs) and long non-coding RNAs (lncRNAs).

NcRNAs operate in various ways on target genes and engage in interactions with one another, forming a complex and dynamic regulatory RNA network. Fluctuations in the expression of a specific ncRNA can influence the expression of other ncRNAs, hence modifying numerous cellular activities, including gene expression, RNA splicing, editing, intracellular transport, and translation. Recent research collectively indicates that miRNAs, piRNAs, endogenous siRNAs, and long non-coding RNAs are the predominant regulatory RNAs. Since our focus is on lncRNAs and miRNAs, we will also describe the function and mechanism of action of these two non-coding RNAs

### 2.1. Long Non-Coding RNAs

Long non-coding RNAs (lncRNAs) are a substantial and very heterogeneous category of non-coding RNAs, exceeding 200 nucleotides in length. The development of high-throughput sequencing technologies has revealed a higher abundance of lncRNAs than previously thought. The GENCODE project has currently annotated more than 16,000 human lncRNAs; however, other studies suggest that the actual number may exceed 100,000 [8]. While many of these lncRNAs are still not well understood, a growing body of evidence indicates that a significant proportion of them play a role in gene regulation and have a biological function [9] (GENCODE v43, 2024; not all transcripts are functionally validated).

Long non-coding RNAs can be transcribed in either sense or antisense orientations from diverse genomic regions, including introns or exons of overlapping protein-coding genes, intergenic regions (lincRNAs), pseudogenes (pseudogene-derived lncRNAs), transcribed ultra conserved regions (T-UCRs), telomeres (telomeric repeat-containing RNAs), centromeric repeats (centromeric lncRNAs), ribosomal DNA loci (promoter and pre-rRNA antisense (PAPAS)), promoters (promoter-associated lncRNAs (PALRs)), enhancers (eRNAs), and 3′ untranslated regions (UTR-associated RNAs).

Like mRNAs, lncRNAs can undergo splicing; however, they typically possess fewer exons, are frequently retained in the nucleus, and their quantity may vary. The diversity of lncRNAs is seen in their functions, which encompass genomic, transcriptional, and translational regulation of both adjacent and distant genes. They can directly engage with DNA and regulate chromatin by building complexes with proteins that attract chromatin modifiers to the promoter regions of their target genes, resulting in the formation of R-loops, and so can bind with enhancers or promoters, thereby activating or inhibiting their functions. These R-loops or triple-helix structures can modulate promoter accessibility, either repressing or activating transcription depending on context [10].

### 2.2. microRNAs

microRNAs (miRNAs) are short non-coding, single-stranded, small RNAs with an average length of 22 nucleotides. These RNA molecules do not encode proteins and are extremely conserved among eukaryotic species. It is anticipated that the majority of the human transcriptome is regulated by miRNAs, playing roles in all essential biological processes, such as cell proliferation, differentiation, and embryonic development, with evidence of their tissue-specific actions established. They regulate post-transcriptional gene expression through protein synthesis inhibition via binding to the untranslated regions (UTRs) of mRNA at the 3′ or 5′ terminal ends. miRNAs often exhibit a higher binding affinity to the 3′ end of their target mRNA sequences, specifically within the 3′ untranslated region (3′UTR). The significance of their crucial function in regulating genes is demonstrated by the estimation that miRNA controls around one-third of human gene expression [11,12]. miRNAs are produced through the enzymatic action of RNA polymerase II or III, which subsequently subjects RNA transcripts to post- or co-transcriptional processing [13]. Despite their lack of protein synthesis capability, miRNAs exert substantial control over an extensive array of biological processes through their involvement in critical pathways and contribution to the regulation of typical animal development.

While miRNAs generally suppress gene expression, there are occasions where they enhance translation. For instance, human miR-369 has been demonstrated to facilitate translation by a method that entails direct interaction with *TNF* and *FXR1*. Furthermore, *let-7 miRNA* has been demonstrated to enhance the translation of its target mRNAs during cell cycle arrest while inhibiting translation in rapidly proliferating cells, suggesting that miRNA functionality oscillates between repression and activation throughout the cell cycle. MiRNAs can also activate genes by attaching to the coding sequence (CDS) or the 5′ untranslated region (UTR) of mRNAs.

Besides controlling transcription in their originating cells, miRNAs can function as intercellular communication molecules by being secreted in extracellular vesicles or by acting as hormones. Additionally, secreted miRNAs can directly engage Toll-like receptors (TLRs) as their ligands, a process that activates TLR signaling transduction pathways and elicits an immunological response.

## 3. Long Noncoding RNAs in Endometriosis

Although functional studies of lncRNAs in endometriosis remain fewer than expression-based analyses, their regulatory roles are increasingly evident [14].

Dysregulated production of lncRNAs in illness can result in symptoms of endometriosis or infertility by impacting the growth, invasion, spread, or transformation of endometrial stromal cells (ESCs). These factors are strongly associated with the progression of endometriosis [15]. LncRNAs have the potential to control the flow of genetic information by influencing many processes such as chromatin structure, transcription, splicing, mRNA stability, mRNA accessibility, and post-translational modifications. The interaction domains for DNA, mRNAs, miRNAs, and proteins are determined by their nucleotide sequence and secondary structure [16].

### 3.1. The Molecular Pathways of lncRNAs in Endometriosis

A recent study has demonstrated that lncRNAs play a critical role in preserving the internal balance of cells or tissues as they develop [17]. Studies have shown that LncRNAs play a significant role in various physiological and pathological processes [18,19].

Three groups of lncRNAs play a role in endometriosis: lncRNAs that attract and direct chromatin remodeling or transcriptional regulatory factors; lncRNAs that act as sponges for miRNAs; lncRNAs that regulate cellular signaling pathways [20].

#### 3.1.1. lncRNAs That Recruit and Target Chromatin Remodeling or Transcriptional Regulatory Factors

Long non-coding RNAs (lncRNAs) are essential in modulating apoptosis by affecting gene expression through epigenetic regulation, transcriptional, and post-transcriptional pathways. At the epigenetic level, lncRNAs can function as scaffolds or guides for chromatin-modifying complexes. Specific sequences or structural motifs may serve as recruiting sites for chromatin remodeling complexes on genomic DNA, thereby modifying chromatin accessibility and influencing gene expression.

At the transcriptional level, lncRNAs can associate with RNA polymerase II (Pol II), thereby limiting transcriptional activity and downregulating gene expression. In certain instances, they can establish triple helix configurations on DNA, enabling direct interaction with gene promoters and affecting the transcriptional apparatus. Moreover, lncRNAs can recruit transcription factors or transcription-associated proteins to target genes, so modulating transcription either positively or negatively based on the context. Post-transcriptionally, lncRNAs regulate mRNA processing. They can affect splicing by engaging with spliceosome components or by binding to complementary antisense lncRNAs at specific mRNA locations, therefore altering the splicing outcome and transcript stability. Through these multifaceted pathways, lncRNAs facilitate the precise regulation of gene expression and are integral to cellular processes such as apoptosis.

Long non-coding RNAs (lncRNAs) are involved in the regulation of apoptosis through epigenetic regulation, transcription, post-transcription, and mediating biological processes; for instance, lncRNAs can bind to RNA polymerase II (Pol II) to inhibit DNA expression via epigenetic regulation; to regulate, they can form a triple helix structure on DNA; and to recruit transcription-related factors or transcription factors to target genes in order to modulate transcription of target genes [21,22,23]. These lncRNA-specific sequences or structures may serve as recruitment sites for the chromatin reconstruction complex on genomic DNA [24,25]. Post-transcriptional regulation affects the binding of the spliceosome and controls the process of shearing mRNA by binding to antisense lncRNA at the specific region of mRNA [26,27].

#### 3.1.2. lncRNAs with miRNA Sponging Functions

**Figure 1:**
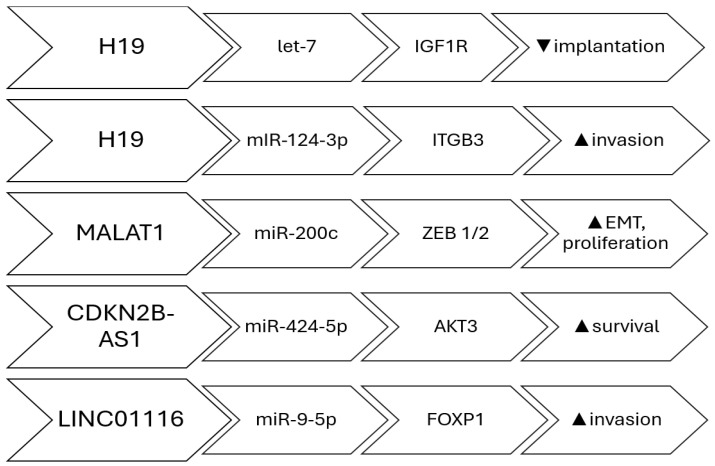
lncRNAs involved in miRNA sponging; Pathways, target and effect.

There is increasing data suggesting that lncRNAs can function as miRNA sponges in endometriosis. In such instances, both the lncRNA and the targeted protein-coding gene contain a binding site for the miRNA, and there exists a link between the expression of the lncRNA and the protein-coding gene. The first documented case of sponging in endometriosis demonstrated a correlation between decreased levels of *H19 lncRNA* and increased activity of *let-7 miRNA*. This increased activity of *let-7 miRNA* inhibits the expression of IGF1R, which leads to a decrease in the proliferation of endometrial stroma cells [28]. These findings indicate that the *H19/let7/IGF1R* pathway may play a role in the reduced ability of the endometrium to support implantation in women with the condition. *H19* has been demonstrated to control the growth and invasion of endometrial cells located outside their normal location by upregulating the expression of *ITGB3* through the sequestration of *miR-124-3p* [29]. *H19* has been linked to compromised immunological responses in women with the condition. It functions as a reservoir for *miR-342-3p*, which controls the *IER3* pathway. This pathway has been linked to the process of Th-17 cell development and the growth of endometrial stroma cells in abnormal locations in women affected by the disease [30]. *CDKN2B-AS1* is an additional long non-coding RNA (lncRNA) that has been demonstrated to function as a sponge in endometriosis. It regulates *AKT3* expression by absorbing miR-424-5p in a laboratory model of ovarian endometriosis, in a primary human endometrial stromal cell culture [31]. *LINC01116* facilitated the growth and movement of endometrial stroma cells by specifically interacting with *FOXP1* through the absorption of *miR-9-5p*. Consequently, this process contributed to the development and expansion of endometriosis lesions [32]. In women with endometriosis, *MALAT1* was discovered to function as a sponge for *miR-200c*. This interaction regulates the proliferation and migration of endometriosis stoma cells by enhancing the expression of *ZEB1* and *ZEB2* [33]. This regulation is not limited to *miR-200c* alone but rather encompasses the full *miR-200* family, which comprises *miR-200a*, *miR-200b*, *miR-200c*, *miR-141*, and *miR-429* [34]. Furthermore, there are other lncRNAs that have been linked to endometriosis due to their function as miRNA sponges. The long non-coding RNAs (lncRNAs) that function as molecular sponges of miRNAs in endometriosis are: *H19* [28], *CDKN2B-AS1* [31], *LINC01541* [35], *LINC01116* [32], *SNHG4* [36], *LINC01018* [37], *SMIM25* [37], *MALAT1* [38], *LINC00261* [39], and *PCAT1* [40] (Figure 1).

#### 3.1.3. lncRNAs That Modulate Cellular Signaling Pathways

**Figure 2:**
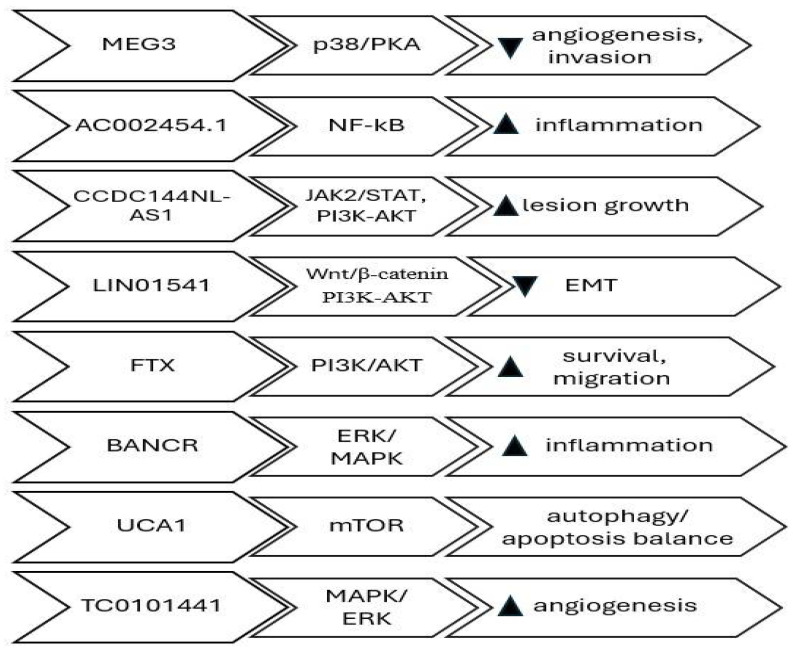
lncRNAs involved in chromatin remodeling, transcription regulation and signal pathway modulation; Pathways, target and effect.

Cell signaling pathways play a crucial role in controlling many cellular processes in response to stimuli from inside or outside the cell. lncRNAs can regulate components of a signaling pathway either directly or indirectly, leading to functional alterations in the signaling cascades. Direct regulation can be accomplished through the direct binding of the long non-coding RNA (lncRNA) to signaling proteins, resulting in alterations in either their abundance within the cell or their functional activity. Indirect regulation refers to situations where there is no proven direct binding of lncRNA to signaling molecules. Instead, it is believed that the lncRNA modifies the transcription of genes related to the signaling pathway, leading to a change in the physiological response. Various cellular signaling pathways impact the regulation of migration, invasion, apoptosis, lesion growth, vascularization, proliferation, ovarian follicle count, infertility, hypoxia-induced pro-survival, autophagy, EMT, angiogenesis, and cell proliferation, thereby promoting the survival and invasion of endometriosis cyst stromal cells (ECSCs). The lncRNAs that regulate cellular signaling pathways are *MEG3-210* (inhibits angiogenesis and invasion by targeting the p38/PKA pathway) [41], *MALAT1* (promotes epithelial–mesenchymal transition and proliferation through the PI3K–AKT axis) [42], *LINC01541* (attenuates the Wnt/β-catenin and TGF-β/Smad axes) [43], *FTX* (activates PI3K/AKT signaling, enhancing survival and migration) [44], *BANCR* (modulates ERK/MAPK signaling, influencing inflammatory cytokine expression) [45], *UCA1* (regulates autophagy and apoptosis balance through the mTOR pathway) [46], *AC002454.1* (activates NF-κB-mediated inflammatory signaling, sustaining cytokine release) [47], *CCDC144NL-AS1-* promotes lesion expansion through STAT3/JAK2 and PI3K-AKT pathways [48], and *TC0101441* (enhances angiogenic and migratory behavior by stimulating the MAPK/ERK cascade) [49] Table 1. Collectively, these lncRNAs act as upstream modulators of cell signaling and may represent diagnostic or therapeutic targets. Studies have demonstrated that phytochemicals such as thymol can modulate these same estrogen-dependent and inflammatory pathways, further emphasizing the importance of hormonal and immune regulation in endometriosis pathophysiology. Thymol has been shown to disrupt PI3K/AKT- and estrogen-mediated inflammatory signaling in endometriotic tissue, illustrating how small-molecule modulation of these same cascades can influence lesion progression [50] (Figure 2).

## 4. microRNAs in Endometriosis

Multiple studies have documented the differential expression of miRNAs in endometrial tissues and extracellular body fluids of women diagnosed with endometriosis as compared to those without the condition [51,52]. This variation in miRNA expression indicates that gene regulation has been disrupted.

### The Pathological Role of miRNAs in Endometriosis

A recent study has revealed the existence of an unusual set of miRNAs that are associated with endometriosis. These miRNAs have a notable impact on the expression of specific target mRNAs. miRNAs have a wide range of functions and play a role in different stages of endometriosis. These compounds demonstrate exceptional stability and can be discovered within cells as well as in different bodily fluids. Their modified manifestation in both blood and endometrial tissues indicates their potential significance in the pathology of endometriosis, as well as infertility linked with endometriosis [53,54,55]. Comparable miRNA-mediated inflammatory mechanisms have also been identified in uterine disorders such as endometritis, where differential expression of key miRNAs similarly governs immune and angiogenic pathways [56]. The overexpression of miRNAs in endometriosis is believed to potentially stimulate the development of the condition. Multiple studies have documented alterations in the expression of certain miRNAs in endometriotic lesions. The miRNAs mentioned include *miR-1*, *miR-29c*, *miR-34c*, *miR-100*, *miR-141*, *miR-145*, *miR-183*, *miR-196b*, *miR-200a*, *miR-200b*, *miR-200c*, *miR-202*, *miR-365*, and *miR-375* [56]. Several of these miRNAs have been identified as being involved in several processes, including Epithelial–Mesenchymal Transition (EMT), angiogenesis, cell proliferation, cell adhesion, and invasion [57,58]. Epithelial–mesenchymal transition (EMT) and angiogenesis play a vital role in the development of endometriotic lesions. Epithelial–mesenchymal transition (EMT) is linked to the movement and infiltration of cells during the formation of lesions, whereas angiogenesis is necessary to construct a vascular network for the expanding lesions. Furthermore, other miRNAs that are not functioning properly are emphasized, such as *miR-15*, *miR-20a*, *miR-23a/b*, *miR-29c*, *miR-126*, *miR-142*, *miR-145*, *miR-183*, *miR-199a*, and *miR-451*. There are differences identified between miRNAs that are likely to be important factors in the disease, affecting the growth of cells, invasion, and the formation of new blood vessels [59] Table 2. The study conducted by Ohlsson Teague et al. examined matched samples of normal and abnormal endometrial tissue in individuals with endometriosis. Microarray analysis identified 22 miRNAs that showed differential expression. Among these, 14 miRNAs were upregulated (*miR 145*, *miR 143*, *miR 99a*, *miR 99b*, *miR 126*, *miR 100*, *miR 125b*, *miR 150*, *miR 125a*, *miR 223*, *miR 194*, *miR 365*, *miR 29c*, and *miR 1*), while 8 miRNAs were downregulated (*miR 200a*, *miR 141*, *miR 200b*, *miR 142 3p*, *miR 424*, *miR 34c*, *miR 20a*, and *miR 196b*). Out of them, miR-145 showed the highest increase in expression, whereas *miR-141* showed the highest decrease [60]. Cho et al. conducted a comparative analysis of miRNA expression in serum samples from women with and without endometriosis. The researchers discovered that the expressions of miRNAs *miR-135b*, *let-7b*, *let-7d*, and *let-7f* was reduced in patients with endometriosis. Their suggestion posits that the involvement of *let-7b* in endometriosis may be linked to the disruption of the p53 pathway and the regulation of cell cycle control [61]. Analysis of peripheral blood samples from women with Stage III and IV endometriosis compared to those without endometriosis identified 27 miRNAs that showed distinct levels of expression. Endometriosis dramatically downregulates six microRNAs: *miR-17-5p*, *miR-20a*, *miR-22*, *miR-15b-5p*, *miR-21*, and *miR-26a*. This study also noted changes in the levels of angiogenesis-related components such as VEGF A and TSP-1 [62,63]. The relevance of miR-20a in endometriosis is still a subject of debate, as several studies have suggested that it is increased in cases of endometriosis [64]. Additional research is needed to better understand the role of this gene in regulating angiogenesis and its contribution to the development of endometriosis. Braza- Boïls et al. discovered a decrease in the expression of miR-449b-3p in ovarian endometriomas when compared to eutopic endometrium. In addition, eutopic endometrial tissue affected by illness had decreased levels of miRNAs *miR-202-3p*, *miR-424-5p*, *miR-449b-3p*, and *miR-556-3p*, while showing increased levels of VEGF A. The data indicates that miRNAs may play a role in modulating angiogenic activity in endometriosis [65]. Zolbin et al. performed a study on adipocyte cells that were genetically modified with miRNA mimics and inhibitors (*let-7b* and *miR-342-3p*). It was discovered that changes in miRNA levels can affect the activity of genes related to the development of brown fat cells, hunger, insulin sensitivity, and fat metabolism. This could potentially explain the low BMI phenotype seen in endometriosis patients [66]. Elevated levels of miR-146 were observed in patients with endometriosis, particularly in those experiencing pain symptoms. These patients also exhibited decreased expression of Interferon Regulatory Factor 5 (*IRF5*), a negative regulator of inflammation. The discovery indicated a significant involvement of the *miR-146b* level and variations in endometriosis [67]. Another independent investigation into miRNA associated with endometriosis revealed that a decreased level of *miR-126-5p*, together with increased *BCAR3* expression, promoted the movement and infiltration of endometriosis stromal cells [68]. Studies demonstrated that the miR-200 family, which plays a vital role in the process of epithelial–mesenchymal transition (EMT) that is essential for the development of endometriotic lesions, exhibited decreased expression in both endometriomas and endometriotic lesions [69,70]. Furthermore, the decrease in *miR-214-3p* expression was associated with the inhibition of endometriosis lesion fibrosis through the targeting of connective tissue growth factor (CCN2) [71]. The decreased expression of *miRNA-34a-5p* in endometrial stem cells resulted in the upregulation of VEGFA, which subsequently stimulated angiogenesis and played a role in the growth and advancement of endometriosis [72]. 

## 5. Conclusions

Despite increasing interest and several discoveries about the involvement of non-coding RNAs in endometriosis, substantial gaps persist in the existing research on both miRNAs and lncRNAs. The research is constrained by small sample sizes, absence of standardized techniques for data collection and analysis, and insufficient replication across separate cohorts, hence limiting the reliability and generalizability of findings. Despite the identification of numerous miRNAs and lncRNAs that are differentially expressed in endometriosis and associated with critical pathophysiological mechanisms, only a limited number have been subjected to thorough functional validation via mechanistic studies in vitro or in vivo. Consequently, the causal links between these non-coding RNAs and disease progression remain ambiguous.

Both miRNAs and lncRNAs have been analyzed in various sample types, including eutopic and ectopic endometrial tissue, serum, peripheral blood, and peritoneal fluid.

They have been identified as differentially expressed in endometriosis and linked to key pathophysiological mechanisms—such as EMT, angiogenesis, oxidative stress, and cell proliferation. However, results across studies are sometimes contradictory. For instance, *miR-451a* is found to be elevated in serum, exosomes, and lesions of endometriosis patients and in animal models, while another study reported decreased *miR-451* expression in normal endometrial tissue of affected individuals. Similarly, *UCA1 lncRNA* was shown to be upregulated in ectopic endometrial tissues by qRT-PCR, but a microarray reported its downregulation in ovarian lesions. Discrepancies were also observed for *MALAT1*, which was increased in endometrial tissues but decreased in granulosa cells of patients with endometriosis.

LncRNAs are known to regulate multiple processes involved in endometriosis development, including stemness, immune response, autophagy, and endometrial receptivity. They can act as miRNA sponges, modulate inflammatory markers, and regulate the proliferation, migration, and apoptosis of endometrial cells, as well as the implantation process. They influence key transcription factors and signaling pathways, including *HOX genes*, *N-cadherin*, *Snail*, *Slug*, *ZEB1*, *MMPs*, *caspases*, and *Beclin1*. Their dysregulated expression may result from genetic predisposition or environmental triggers, and while transcriptomic studies have identified many candidate lncRNAs, only a limited number have been validated. Challenges remain due to small cohorts, lack of well-characterized clinical samples, and inconsistent control groups. Most existing studies are confined to in vitro analyses, and there is a lack of robust in vivo evidence supporting the clinical applicability of lncRNAs for diagnosis or treatment. While many lncRNAs show potential as therapeutic targets, efficient and reliable testing methods for clinical implementation are still lacking.

MiRNAs, which regulate gene expression post-transcriptionally, form complex regulatory networks where one miRNA can target multiple mRNAs, and vice versa. Their tissue-specific expression and involvement in endometriosis-related processes such as adhesion, proliferation, angiogenesis, and cell death have been widely documented. However, miRNA profiles in endometriosis and related disorders like recurrent implantation failure often overlap, and inconsistencies in results across studies hinder clear clinical translation.

In conclusion, extensive genomic and transcriptomic investigations have linked numerous long non-coding RNAs (lncRNAs) and microRNAs to endometriosis. The task at hand is to differentiate the lncRNAs and microRNAs that have a significant impact on the disease from those that are only linked to the changes in gene expression in the disease. Additionally, it is important to demonstrate their functional relevance.

## Figures and Tables

**Table 1:** Differentially expressed lncRNAs implicated in endometriosis.

lncRNA	Mechanism/Target	Biological Role
H19	Regulates IGF1R via let-7 sponging	Reduces stromal proliferation
CDKN2B-AS1	Regulates AKT3 by binding miR-424-5p	Promotes invasion in ovarian endometriosis
LINC01116	Targets FOXP1 via miR-9-5p sponging	Promotes stromal cell proliferation and migration
MALAT1	Epigenetic regulation through ZEB1/ZEB2 activation	Enhances EMT and migration
LINC01541	Wnt/β-catenin and TGF-β/Smad	Reduces EMT and proliferation
AC002454.1	NF-κB signaling	Promotes inflammation and cytokine release
CCDC144NL-AS1	STAT3/JAK2 and PI3K-AKT	Enhances lesion proliferation
TC0101441	MAPK/ERK cascade	Promotes angiogenesis and migration
MEG3-210	p38/PKA pathway	Inhibits angiogenesis and invasion
FTX	PI3K/AKT	Supports proliferation and survival
BANCR	ERK/MAPK	Regulates inflammatory cytokines
UCA1	mTOR axis	Balances autophagy and apoptosis

**Table 2:** Differentially expressed miRNAs implicated in endometriosis.

miRNA	Target/Pathway	Biological Effect
miR-145	Cytoskeletal and proliferation genes	Inhibits proliferation
miR-200 family	ZEB1/ZEB2 (EMT regulators)	Suppresses EMT
miR-146b	IRF5	Modulates inflammation and pain
miR-126-5p	BCAR3	Enhances cell migration/invasion
miR-34a-5p	VEGFA	Regulates angiogenesis
miR-214-3p	CCN2	Reduces fibrosis
let-7b	p53 pathway	Regulates cell cycle
miR-20a	ERK and angiogenesis genes	Alters angiogenesis

## Data Availability

Not applicable.

## Abbreviations

The following abbreviations are used in this manuscript:

lncRNAs	Long non-coding RNAs
miRNAs	Micro RNAs
EMT	Epithelial–Mesenchymal Transition
Pol II	RNA Polymerase II
VEGF A	Vascular endothelial growth factor A
MALAT1	Metastasis-associated lung adenocarcinoma transcript 1
ESCs	Endometrial stromal cells
ZEB1	Zinc finger E-box-binding homeobox 1
ncRNAs	Noncoding RNAs

## References

[1] Rogers P.A., D‘Hooghe T.M., Fazleabas A., Giudice L.C., Montgomery G.W., Petraglia F., Taylor R.N. (2013). Defining future directions for endometriosis research: Workshop report from the 2011 World Congress of Endometriosis In Montpellier, France. Reprod. Sci..

[2] Bulletti C., Coccia M.E., Battistoni S., Borini A. (2010). Endometriosis and infertility. J. Assist. Reprod. Genet..

[3] Burney R.O., Giudice L.C. (2012). Pathogenesis and pathophysiology of endometriosis. Fertil. Steril..

[4] Prapas Y., Goudakou M., Matalliotakis I., Kalogeraki A., Matalliotaki C., Panagiotidis Y., Ravanos K., Prapas N. (2012). History of endometriosis may adversely affect the outcome in menopausal recipients of sibling oocytes. Reprod. Biomed. Online.

[5] Becker C.M., Bokor A., Heikinheimo O., Horne A., Jansen F., Kiesel L., King K., Kvaskoff M., Nap A., Petersen K. (2022). ESHRE guideline: Endometriosis. Hum. Reprod. Open.

[6] Kuznetsov L., Dworzynski K., Davies M., Overton C., Guideline Committee (2017). Diagnosis and management of endometriosis: Summary of NICE guidance. BMJ.

[7] Panir K., Schjenken J.E., Robertson S.A., Hull M.L. (2018). Non-coding RNAs in endometriosis: A narrative review. Hum. Reprod. Update.

[8] Statello L., Guo C.J., Chen L.L., Huarte M. (2021). Gene regulation by long non-coding RNAs and its biological functions. Nat. Rev. Mol. Cell Biol..

[9] Gil N., Ulitsky I. (2020). Regulation of gene expression by cis-acting long non-coding RNAs. Nat. Rev. Genet..

[10] Crossley M.P., Bocek M., Cimprich K.A. (2019). R-Loops as Cellular Regulators and Genomic Threats. Mol. Cell.

[11] Lu T.X., Rothenberg M.E. (2018). MicroRNA. J. Allergy Clin. Immunol..

[12] Correia de Sousa M., Gjorgjieva M., Dolicka D., Sobolewski C., Foti M. (2019). Deciphering miRNAs’ Action through miRNA Editing. Int. J. Mol. Sci..

[13] O’Brien J., Hayder H., Zayed Y., Peng C. (2018). Overview of MicroRNA Biogenesis, Mechanisms of Actions, and Circulation. Front. Endocrinol..

[14] Delás M.J., Hannon G.J. (2017). lncRNAs in development and disease: From functions to mechanisms. Open Biol..

[15] Wang M., Zheng L., Lin R., Ma S., Li J., Yang S. (2023). A comprehensive overview of exosome lncRNAs: Emerging biomarkers and potential therapeutics in endometriosis. Front. Endocrinol..

[16] Fernandes J.C.R., Acuña S.M., Aoki J.I., Floeter-Winter L.M., Muxel S.M. (2019). Long non-coding RNAs in the regulation of gene expression: Physiology and disease. Noncoding RNA.

[17] Maligianni I., Yapijakis C., Nousia K., Bacopoulou F., Chrousos G.P.P. (2022). Exosomes and exosomal noncoding RNAs throughout human gestation (Review). Exp. Ther. Med..

[18] Zhang T., Tang X., Zhu Y., Wang C., Jiang Z., Yang N., Wang T., Shu L., Xu Y., Sun L. (2023). IGF2BP2 enhances LincRNA01116 stability via m^6^ A: A potential biomarker and therapeutic target for patients with preeclampsia. J. Cell Biochem..

[19] Ghafouri-Fard S., Shoorei H., Taheri M. (2020). Role of noncoding RNAs in the pathogenesis of endometriosis. Front. Oncol..

[20] Hudson Q.J., Proestling K., Perricos A., Kuessel L., Husslein H., Wenzl R., Yotova I. (2021). The Role of Long Non-Coding RNAs in Endometriosis. Int. J. Mol. Sci..

[21] Nunez-Martinez H.N., Recillas-Targa F. (2022). Emerging functions of lncRNA loci beyond the transcript itself. Int. J. Mol. Sci..

[22] Yang L., Li L.-P., Yi H.-C. (2022). DeepWalk-based method to predict lncRNA-miRNA associations via lncRNA-miRNA-disease-protein-drug graph. BMC Bioinf..

[23] Zhao W., Liu Y., Zhang C., Duan C. (2019). Multiple roles of exosomal long noncoding RNAs in cancers. BioMed Res. Int..

[24] Huang W., Li H., Yu Q., Xiao W., Wang D.O. (2022). LncRNA-mediated DNA methylation: An emerging mechanism in cancer and beyond. J. Exp. Clin. Cancer Res..

[25] Herman A.B., Tsitsipatis D., Gorospe M. (2022). Integrated lncRNA function upon genomic and epigenomic regulation. Mol. Cell.

[26] Sanbonmatsu K. (2022). Getting to the bottom of lncRNA mechanism: Structure-function relationships. Mamm. Genome.

[27] Kopp F., Mendell J.T. (2018). Functional classification and experimental dissection of long noncoding RNAs. Cell.

[28] Ghazal S., McKinnon B., Zhou J., Mueller M., Men Y., Yang L., Mueller M., Flannery C., Huang Y., Taylor H.S. (2015). H19 lncRNA alters stromal cell growth via IGF signaling in the endometrium of women with endometriosis. EMBO Mol. Med..

[29] Liu S., Qiu J., Tang X., Cui H., Zhang Q., Yang Q. (2019). LncRNA-H19 regulates cell proliferation and invasion of ectopic endometrium by targeting ITGB3 via modulating miR-124-3p. Exp. Cell Res..

[30] Liu Z., Liu L., Zhong Y., Cai M., Gao J., Tan C., Han X., Guo R., Han L. (2019). LncRNA H19 over-expression inhibited Th17 cell differentiation to relieve endometriosis through miR-342-3p/IER3 pathway. Cell Biosci..

[31] Wang S., Yi M., Zhang X., Zhang T., Jiang L., Cao L., Zhou Y., Fang X. (2021). Effects of CDKN2B-AS1 on cellular proliferation, invasion and AKT3 expression are attenuated by miR-424-5p in a model of ovarian endometriosis. Reprod. Biomed. Online.

[32] Cui L., Chen S., Wang D., Yang Q. (2021). LINC01116 promotes proliferation and migration of endometrial stromal cells by targeting FOXP1 via sponging miR-9-5p in endometriosis. J. Cell Mol. Med..

[33] Liang Z., Chen Y., Zhao Y., Xu C., Zhang A., Zhang Q., Wang D., He J., Hua W., Duan P. (2017). miR-200c suppresses endometriosis by targeting MALAT1 in vitro and in vivo. Stem Cell Res. Ther..

[34] Du Y., Zhang Z., Xiong W., Li N., Liu H., He H., Li Q., Liu Y., Zhang L. (2019). Estradiol promotes EMT in endometriosis via MALAT1/miR200s sponge function. Reproduction.

[35] Xu Z., Zhang L., Yu Q., Zhang Y., Yan L., Chen Z.J. (2019). The estrogen-regulated lncRNA H19/miR-216a-5p axis alters stromal cell invasion and migration via ACTA2 in endometriosis. Mol. Hum. Reprod..

[36] Mai H., Xu H., Lin H., Wei Y., Yin Y., Huang Y., Huang S., Liao Y. (2021). LINC01541 Functions as a ceRNA to Modulate the Wnt/beta-Catenin Pathway by Decoying miR-506-5p in Endometriosis. Reprod. Sci..

[37] Jiang L., Zhang M., Wang S., Xiao Y., Wu J., Zhou Y., Fang X. (2020). LINC01018 and SMIM25 sponged miR-182-5p in endometriosis revealed by the ceRNA network construction. Int. J. Immunopathol. Pharmacol..

[38] Liu Y., Huang X., Lu D., Feng Y., Xu R., Li X., Yin C., Xue B., Zhao H., Wang S. (2020). LncRNA SNHG4 promotes the increased growth of endometrial tissue outside the uterine cavity via regulating c-Met mediated by miR-148a-3p. Mol. Cell Endocrinol..

[39] Feng Y., Tan B.Z. (2020). LncRNA MALAT1 inhibits apoptosis of endometrial stromal cells through miR-126-5p-CREB1 axis by activating PI3K-AKT pathway. Mol. Cell Biochem..

[40] Wang L., Xing Q., Feng T., He M., Yu W., Chen H. (2020). SNP rs710886 A > G in long noncoding RNA PCAT1 is associated with the risk of endometriosis by modulating expression of multiple stemness-related genes via microRNA-145 signaling pathway. J. Cell Biochem..

[41] Wang H., Sha L., Huang L., Yang S., Zhou Q., Luo X., Shi B. (2019). LINC00261 functions as a competing endogenous RNA to regulate BCL2L11 expression by sponging miR-132-3p in endometriosis. Am. J. Transl. Res..

[42] Liu Y., Ma J., Cui D., Fei X., Lv Y., Lin J. (2020). LncRNA MEG3-210 regulates endometrial stromal cells migration, invasion and apoptosis through p38 MAPK and PKA/SERCA2 signalling via interaction with Galectin-1 in endometriosis. Mol. Cell Endocrinol..

[43] Liu H., Zhang Z., Xiong W., Zhang L., Du Y., Liu Y., Xiong X. (2019). Long non-coding RNA MALAT1 mediates hypoxia-induced pro-survival autophagy of endometrial stromal cells in endometriosis. J. Cell Mol. Med..

[44] Mai H., Wei Y., Yin Y., Huang S., Lin H., Liao Y., Liu X., Chen X., Shi H., Liu C. (2019). LINC01541 overexpression attenuates the 17beta-Estradiol-induced migration and invasion capabilities of endometrial stromal cells. Syst. Biol. Reprod. Med..

[45] Qiu J.J., Lin X.J., Zheng T.T., Tang X.Y., Zhang Y., Hua K.Q. (2019). The Exosomal Long Noncoding RNA aHIF is Upregulated in Serum from Patients with Endometriosis and Promotes Angiogenesis in Endometriosis. Reprod. Sci..

[46] Yotova I., Hudson Q.J., Pauler F.M., Proestling K., Haslinger I., Kuessel L., Perricos A., Husslein H., Wenzl R. (2021). LINC01133 Inhibits Invasion and Promotes Proliferation in an Endometriosis Epithelial Cell Line. Int. J. Mol. Sci..

[47] Wang H., Ni C., Xiao W., Wang S. (2020). Role of lncRNA FTX in invasion, metastasis, and epithelial-mesenchymal transition of endometrial stromal cells caused by endometriosis by regulating the PI3K/Akt signaling pathway. Ann. Transl. Med..

[48] Zhu M.B., Chen L.P., Hu M., Shi Z., Liu Y.N. (2019). Effects of lncRNA BANCR on endometriosis through ERK/MAPK pathway. Eur. Rev. Med. Pharmacol. Sci..

[49] Jiang L., Wan Y., Feng Z., Liu D., Ouyang L., Li Y., Liu K. (2020). Long Noncoding RNA UCA1 Is Related to Autophagy and Apoptosis in Endometrial Stromal Cells. Front. Oncol..

[50] Zhang Y., Shaukat A., Zhang H., Yang Y.F., Li H.X., Li G.Y., Liu Y.N., Liang C., Kang J.W., Li S.C. (2024). Thymol Impacts the Progression of Endometriosis by Disrupting Estrogen Signaling Pathways and Inflammatory Responses. Int. J. Mol. Sci..

[51] Bjorkman S., Taylor H.S. (2019). MicroRNAs in endometriosis: Biological function and emerging biomarker candidates. Biol. Reprod..

[52] Zhang Y., Zhang H., Yan L., Liang G., Zhu C., Wang Y., Ji S., He C., Sun J., Zhang J. (2023). Exosomal microRNAs in tubal fluid may be involved in damage to tubal reproductive function associated with tubal endometriosis. Reprod. Biomed. Online.

[53] Teague E.M., Print C.G., Hull M.L. (2010). The role of microRNAs in endometriosis and associated reproductive conditions. Hum. Reprod. Update.

[54] Weber J.A., Baxter D.H., Zhang S., Huang D.Y., How Huang K., Jen Lee M., Galas D.J., Wang K. (2010). The microRNA spectrum in 12 body fluids. Clin. Chem..

[55] Fehlmann T., Ludwig N., Backes C., Meese E., Keller A. (2016). Distribution of microRNA biomarker candidates in solid tissues and body fluids. RNA Biol..

[56] Wang S., Cao Z., Wu Q., Dong H. (2023). A Comparative Analysis and Verification of Differentially Expressed MiRNAs Could Provide New Insights for the Treatment of Endometritis in Yaks. Pak. Vet. J..

[57] Antonio L.G.L., Meola J., Rosa-e-Silva A.C.J.d.S., Nogueira A.A., Candido dos Reis F.J., Poli-Neto O.B., Rosa-e-Silva J.C. (2023). Altered Differential Expression of miRNAs Related to Adhesion and Apoptosis Pathways in Patients with Different Phenotypes of Endometriosis. Int. J. Mol. Sci..

[58] Braicu O.-L., Budisan L., Buiga R., Jurj A., Achimas-Cadariu P., Pop L., Braicu C., Irimie A., Berindan-Neagoe I. (2017). miRNA expression profiling in formalin-fixed paraffin-embedded endometriosis and ovarian cancer samples. OncoTargets Ther..

[59] Nothnick W.B. (2017). MicroRNAs and endometriosis: Distinguishing drivers from passengers in disease pathogenesis. Semin. Reprod. Med..

[60] Ohlsson Teague E.M., Van der Hoek K.H., Van der Hoek M.B., Perry N., Wagaarachchi P., Robertson S.A., Print C.G., Hull L.M. (2009). MicroRNA-regulated pathways associated with endometriosis. Mol. Endocrinol..

[61] Cho S., Mutlu L., Grechukhina O., Taylor H.S. (2015). Circulating microRNAs as potential biomarkers for endometriosis. Fertil. Steril..

[62] Jia S.-Z., Yang Y., Lang J., Sun P., Leng J. (2013). Plasma miR-17-5p, miR-20a and miR-22 are down-regulated in women with endometriosis. Hum. Reprod..

[63] Ramón L.A., Braza-Boïls A., Gilabert-Estellés J., Gilabert J., España F., Chirivella M., Estellés A. (2011). MicroRNAs expression in endometriosis and their relation to angiogenic factors. Hum. Reprod..

[64] Lin S.C., Wang C.C., Wu M.H., Yang S.H., Li Y.H., Tsai S.J. (2012). Hypoxia-induced microRNA-20a expression increases ERK phosphorylation and angiogenic gene expression in endometriotic stromal cells. J. Clin. Endocrinol. Metab..

[65] Braza-Boïls A., Marí-Alexandre J., Gilabert J., Sánchez-Izquierdo D., España F., Estellés A., Gilabert-Estellés J. (2014). MicroRNA expression profile in endometriosis: Its relation to angiogenesis and fibrinolytic factors. Hum. Reprod..

[66] Goetz L.G., Mamillapalli R., Taylor H.S. (2016). Low body mass index in endometriosis is promoted by hepatic metabolic gene dysregulation in mice. Biol. Reprod..

[67] Zhang Z., Li H., Zhao Z., Gao B., Meng L., Feng X. (2019). miR-146b level and variants is associated with endometriosis related macrophages phenotype and plays a pivotal role in the endometriotic pain symptom. Taiwan. J. Obstet. Gynecol..

[68] Meng X., Liu J., Wang H., Chen P., Wang D. (2019). MicroRNA-126-5p downregulates BCAR3 expression to promote cell migration and invasion in endometriosis. Mol. Cell. Endocrinol..

[69] Yang Y.M., Yang W.X. (2017). Epithelial-to-mesenchymal transition in the development of endometriosis. Oncotarget.

[70] Viganò P., Ottolina J., Bartiromo L., Bonavina G., Schimberni M., Villanacci R., Candiani M. (2020). Cellular components contributing to fibrosis in endometriosis: A literature review. J. Minim. Invasive Gynecol..

[71] Zhang Y., Chang X., Wu D., Deng M., Miao J., Jin Z. (2020). Down-regulation of exosomal miR-214-3p targeting CCN2 contributes to endometriosis fibrosis and the role of exosomes in the horizontal transfer of miR-214-3p. Reprod. Sci..

[72] Ma Y., Huang Y.-X., Chen Y.-Y. (2017). miRNA-34a-5p downregulation of VEGFA in endometrial stem cells contributes to the pathogenesis of endometriosis. Mol. Med. Rep..

